# Mechanical Properties and Cytotoxicity of Differently Structured Nanocellulose-hydroxyapatite Based Composites for Bone Regeneration Application

**DOI:** 10.3390/nano10010025

**Published:** 2019-12-20

**Authors:** Vijay H. Ingole, Tomaž Vuherer, Uroš Maver, Aruna Vinchurkar, Anil V. Ghule, Vanja Kokol

**Affiliations:** 1 Department of Nanotechnology, Dr. Babasaheb Ambedkar Marathwada University, Aurangabad 431004, Maharashtra, India; mvt.vijay@gmail.com (V.H.I.); anighule@gmail.com (A.V.G.); 2 Institute of Engineering Materials and Design, Faculty of Mechanical Engineering, University of Maribor, Smetanova ulica 17, Maribor SI-2000, Slovenia; tomaz.vuherer@um.si; 3 Institute of Biomedical Sciences and Department of Pharmacology, Faculty of Medicine, University of Maribor, Taborska ulica 8, Maribor SI-2000, Slovenia; uros.maver@um.si; 4 Department of Biophysics, Government Institute of Science, Dr. Babasaheb Ambedkar Marathwada University, Aurangabad 431004, Maharashtra, India; aruna.vinchurkar@gmail.com; 5 Department of Chemistry, Shivaji University, Kolhapur 416004, Maharashtra, India

**Keywords:** nanocomposites, nanocellulose, hydroxyapatite, mechanical properties, cytotoxicity

## Abstract

The nanocomposites were prepared by synthesizing (2,2,6,6-tetramethylpiperidin-1-yl)oxyl (TEMPO)-oxidized cellulose nanofibrils (TCNFs) or cellulose nanocrystals (CNCs) with hydroxyapatite (HA) in varying composition ratios in situ. These nanocomposites were first obtained from eggshell-derived calcium and phosphate of ammonium dihydrogen orthophosphate as precursors at a stoichiometric Ca/P ratio of 1.67 with ultrasonication and compressed further by a uniaxial high-pressure technique. Different spectroscopic, microscopic, and thermogravimetric analyses were used to evaluate their structural, crystalline, and morphological properties, while their mechanical properties were assessed by an indentation method. The contents of TCNF and CNC were shown to render the formation of the HA crystallites and thus influenced strongly on the composite nanostructure and further on the mechanical properties. In this sense, the TCNF-based composites with relatively higher contents (30 and 40 wt %) of semicrystalline and flexible TCNFs resulted in smoother and more uniformly distributed HA particles with good interconnectivity, a hardness range of 550–640 MPa, a compression strength range of 110–180 MPa, an elastic modulus of ~5 GPa, and a fracture toughness value of ~6 MPa^1/2^ in the range of that of cortical bone. Furthermore, all the composites did not induce cytotoxicity to human bone-derived osteoblast cells but rather improved their viability, making them promising for bone tissue regeneration in load-bearing applications.

## 1. Introduction

Natural bone is a highly complex and hierarchically structured composite of strong calcium phosphate (mainly hydroxyapatite (HA))-based crystalline phase embedded in a soft organic matrix of collagen proteins and growth factors, thus forming a structure with unique histology and mechanical properties (strength, resilience, and flexibility) [1]. Depending on the degree of fracture, it has significantly, at par, self-repairing capability. Minor fractures usually heal perfectly, while larger fractures, such as segmental bone defects resulting from bone tumor resections or severe nonunionized fractures, often result in permanent damage. The latter can lead to extensive traumatic fractures, osteosarcoma, congenital malformation, or even a hindered regenerative capacity [2]. Currently, autografts containing patient living cells and growth factors are commonly used, although these are far from optimal, especially due to the lack of bone availability and the risk of infections and geometric dissimilarity between a bone and a defect site, altogether often resulting in a rather poor integration [3,4]. However, allografts may be immunogenic and hence rejected by the body [4]. Repairing of larger bone defects thus poses a major challenge [5], which has demanded the development of various synthetic alternative composites during the recent decade. 

Calcium phosphate-based bioceramic materials, particularly HA with a crystalline structure similar to that of a bone, have been shown as an excellent bioactive, resorbable and osteoconductive material to promote bone regeneration [6]. The major limitation of HA is its inherent brittle nature that accounts for low fracture toughness (0.8–1.2 MPa^1/2^) and flexural strength (<140 MPa), which makes their processing into more complex shapes very difficult, hence limiting their use to nonload bearing application only [6]. Since a bone consists of an inorganic–organic phase, designing a synthetic bone-regenerated composite should also be a combination of the advantages of both the types of materials to bring an excellent balance between strength and toughness. Combinations of calcium phosphate (or HA) with various organic polymers, for instance alginate [7], gelatin [8], collagen [9], chitosan [10], starch [11], and bacterial cellulose [12], have thus been utilized to develop artificial bone composites. However, inadequate biomechanical and biological properties, insufficient intrinsic incorporation, and sometimes masking of an inorganic component by an organic one [13], together with a poor dispersibility in a biopolymeric matrix, are still the key drawbacks of the existing formulations. The need for further compositional and structural advancement to enrich these materials with more complex and in vivo improved functionalities is thus still an increasing demand.

Recently, a great deal of attention has been directed towards the development of novel cost-effective tissue-mimicking composites using natural materials and green approaches of their engineering [14]. Nanocellulose, generally classified as either highly crystalline (54–88%) cellulose nanocrystals (CNCs) or semicrystalline cellulose nanofibrils (CNFs), derived from plant biomass, marine animals, or microbial/bacterial sources [15,16,17,18], has been thus also confirmed as promising for various biomedical applications [19,20], due to its large surface area, good biomechanical properties (Young’s modulus up to ~145 GPa and mechanical strength up to ~7500 MPa), abundant hydroxyl groups for potential functionalization, high biocompatibility [21], and insignificant cytotoxicity [22] within the tolerance limit of an immune system [23,24]. Relatively more flexible CNFs with a few micrometer in length and up to 150 nm in diameter have been explored extensively in wound healing [25], tissue engineering [26], and cell therapy [27], while smaller and more rigid CNCs with <300 nm in length and around 10 nm in diameter were used as targeted drugs [28,29,30,31] and genes [32] in delivery systems and in diagnostics [33]. Some studies have been also already reported the bone cell growth and differentiation-promoting effect of bacterial nanocellulose by in vitro [34] experiments, which in a mixture with HA accelerated new bone formation at defect sites in rat tibiae four weeks after implanting [35]. However, there is only a very limited number of studies reporting the use of plant-derived nanocellulose in bone tissue regeneration. Recent examples include porous bioactive glass CNC-based scaffolds, which were found to be bioactive and biocompatible against osteoblast cells [36], as well as porous CNF-based scaffolds [37,38,39] with a positive chondrocyte cell response and a potential in cartilage tissue engineering. In addition, oxidized CNF products have been studied to increase its degradability in vivo [24] and to promote its gradual replacement with an extracellular matrix. In line with this, (2,2,6,6-tetramethylpiperidin-1-yl)oxyl (TEMPO)-oxidized CNFs [40] (TCNFs) was further functionalized with osteoinductive phosphonates [41] to promote osteogenic differentiation of mesenchymal stem cells and increase Ca deposition with the formation of HA-like structures. 

In recent years, sonochemical preparation of narrow-size-distribution nanoparticles [42] (including HA [43,44], calcium phosphate [45], and zinc phosphate [46] has become a promising strategy. It has also been shown suitable for the preparation of cellulose-based nanocomposites, such as cellulose/CaCO_3_ [47] and cellulose/Mn_3_O [48], compared with microwave-assisted or oil heating methods [49,50]. However, the application of a sonochemical method in the preparation of nanocellulose/HA-based composites has not yet been reported. The evaluation of such composites for bone regeneration has also not been taken into consideration yet, and especially the fibrillar nature of CNFs has not been considered, which can mimic natural bone architecture and, as such, initiate and/or enhance osteogenic differentiation [51].

**Figure 1:**
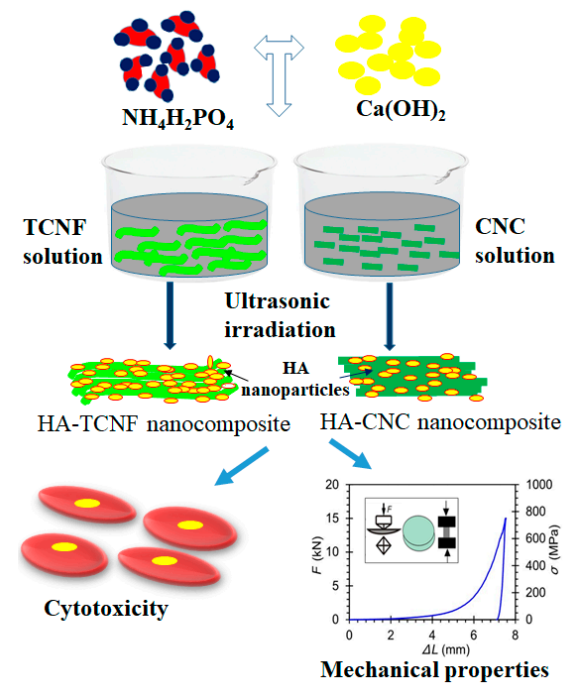
Schematic illustration of nanocellulose/hydroxyapatite (HA) composites preparation by a sonochemical method, followed by mechanical and cytotoxicity testing.

The objective of the present study was to fabricate nanocomposites from different types of nanocellulose (flexible TCNF and rigid CNC) and HA synthesized in situ by a sonochemical method using an eggshell as a derivative of calcium and dihydrogen orthophosphate precursors, as illustrated schematically in Figure 1. The as-prepared nanocomposite powders were further compressed by a uniaxial compression technique and characterized with regards to their morphological, physicochemical, thermal, and mechanical properties. Varying concentrations of the TCNF and the CNC were applied in order to verify the potential of different formulations to mimic an inorganic–organic structure and mechanical properties of a bone. Finally, their cytotoxicity was evaluated by using human bone-derived osteoblast cells.

## 2. Results and Discussion

### 2.1. Structure, Morphology, and Thermal Stability of the Nanocomposites

**Figure 2:**
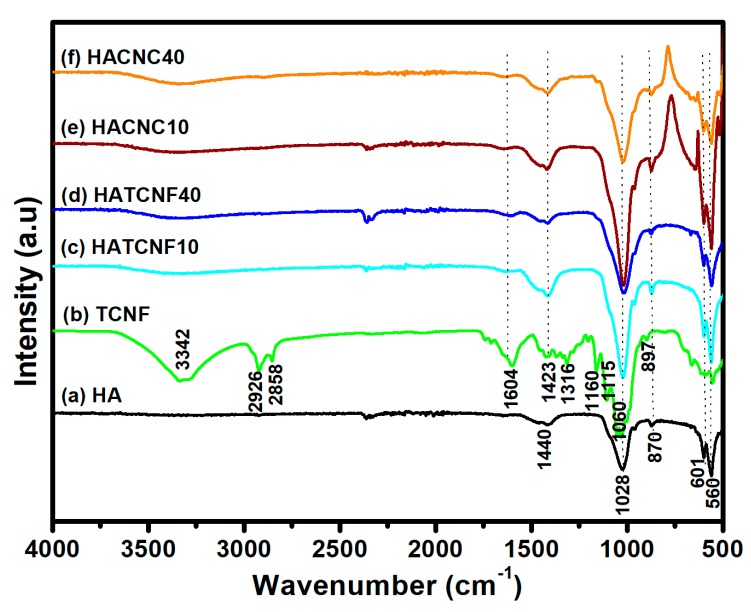
Representative FTIR spectra of the pure HA (**a**) and the (2,2,6,6-tetramethylpiperidin-1-yl)oxyl (TEMPO)-oxidized cellulose nanofibrils (TCNFs) (**b**). FTIR spectra of the corresponding nanocomposites: (**c**) HATCNF10 prepared with 10 wt % of TCNF; (**d**) HATCNF40 prepared with 40 wt % of TCNF; (**e**) HACNC10 prepared with 10 wt % of CNC; and (**f**) HACNC40 prepared with 40 wt % of CNC.

The FTIR spectra (Figure 2) of the HA–TCNF/CNC composites demonstrate typical spectral lines for the HA and the cellulose. The spectrum of the synthesized HA (Figure 2a) reveals specific peaks at around 560 and 601 cm^–1^, corresponding to the phosphate group (PO_4_^3–^) bending vibration [52,53]. The intense and sharp absorption band observed at around 1028 cm^–1^ resembled the symmetric and asymmetric stretching of phosphate, while the bands observed at around 870 and 1440 cm^–1^ originated from the carbonate groups (CO_3_^2–^) in the crystal lattice of the HA. Representative cellulose vibration bands were also identified within the TCNF spectral line as shown in Figure 2b [54]. The glucose ring related to the C–O bond at around 1060 cm^–1^, the –OH vibrations region at around 3342 cm^−1^, and the C–H stretching vibrations at around 2926 cm^−1^, as well as the antisymmetric bridge of the C–O–C stretching and the skeletal vibrations of the C–O stretching at around 1160 and 1115 cm^−1^, respectively. The peaks at around 1316 and 1423 cm^−1^ corresponded to the presence of the –CH_2_ rocking and bending groups [55], while the stronger peak at around 1604 cm^−1^ was associated to the carboxylic (COO^–^) stretching vibrations groups [41,56]. 

In the case of the nanocomposites spectra (Figure 2c–f), the absence or very weak cellulose peaks were observed at around 3342, 2926, 1604, 1316, 1160, and 1060 cm^–1^, indicating that the surfaces of the nanocellulose were probably completely covered with the HA crystals [53]. In addition, increases of the bands at around 1440 cm^–1^ and 870 cm^–1^ corresponding to the carbonate (CO_3_^2–^) groups in the crystal lattice of the HAp [57], being shifted slightly to around 1423 and 897 cm^–1^, respectively, and being more intensive in the case of the nanocomposites with a lower nanocellulose content (HACNC10 and HATCNF10), may present an ionic substitute for PO_4_^3−^ in the apatite crystal of the HA. At the same time, a more intensive band was observed at around 941 cm^–1^ corresponding to the symmetric stretching mode of the P–O of the phosphate group, and peaks at around 560 and 601 cm^–^^1^ corresponded to the PO_4_^3–^ bending of the HA. 

Earlier reports explained that minerals are formed on CNFs due to the adsorption of mineral ions from solutions [34], suggesting that polar functional groups, such as OH^–^ on cellulose backbones or even COO^–^ and OSO_3_^–^ groups in the case of TCNF and CNC, respectively, trigger the nucleation of calcium phosphate crystals [58]. However, other studies [59,60] reflect that cellulose OH^–^ groups do not have a high enough reactivity to grow calcium phosphate crystals, due to significantly weak interactions between negative partial atomic charges of oxygen atoms of OH^–^ groups and positive calcium ions (ion–polar interactions) that probably cannot compensate for entropy losses. 

**Figure 3:**
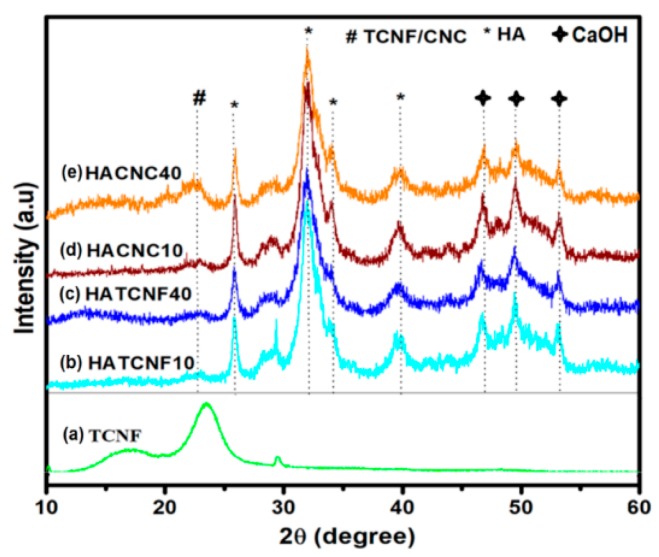
Representative XRD spectra: the pure HA (**a**) and the TCNF (**b**). XRD spectra of the corresponding nanocomposites: (**c**) HATCNF10 prepared with 10 wt % of TCNF; (**d**) HATCNF40 prepared with 40 wt % of TCNF; (**e**) HACNC10 prepared with 10 wt % of CNC; and (**f**) HACNC40 prepared with 40 wt % of CNC.

The XRD analysis of the pure TCNF, as well as those of the HA–TCNF/CNC nanocomposites, was performed to identify the HA morphological and crystalline structure. The XRD spectra of the TCNF (Figure 3a) exhibited typical diffraction peaks at 2θ of around 16° and 22.6°, corresponding to the (101) and (002) planes of cellulose I allomorphs, respectively, representing their partial crystalline nature [54]. On the other hand, the XRD patterns of HA–TCNF/CNC nanocomposites (Figure 3b–e) showed a weak peak at 2θ of around 22.6°, which was characteristic for the cellulose (Figure 3a), and relatively sharp peaks at 2θ between 25° and 35° corresponding to the HA with the Miller indices (hkl) at the (201), (211), (300), and (310) planes, according to the JCPDS-861199 [44,53] database. The lower peaks in the composites (Figure 3b–e) were observed for Ca(OH)_2_ (according to the JCPDS-841276 database) at around 46.9°, 49.4°, and 53.1°. Besides, the composites prepared with 10 and 40 wt % of nanocellulose may be constituted as a mixture of Ca_10_(PO_4_)_6_(OH)_2_ and Ca(OH)_2_, according to the JCPDS-861199 and JCPDS 841276 databases, respectively. In addition, the spectra of the composites prepared with a lower (10 wt %) nanocellulose content resembled the peaks of the synthesized HA with 14.56 nm (HATCNF10) or (16.25 nm (HACNC10) average crystallite sizes in a nonstoichiometric Ca/P structure (Ca/P ratio = 2.35–2.24) (Table 1). On the other hand, the composites prepared with a higher (40 wt %) nanocellulose content showed broader HA-related peaks at 2θ of around 32°–33°, being reflected in the HA with poor crystallinity and lower crystal sizes (12.22–12.53 nm). This effect was expressed more in the case of the composite prepared with 40 wt % of CNC, where the greatest deviation from a stoichiometric Ca/P structure, i.e., calcium-rich (phosphorus-deficient) structure with a Ca/P ratio of 2.48, was identified. In addition to the competitive interactions of mineral ions with the CNF or CNC surface, the reason for such a deviation in a Ca/P value may also be from the replacement of PO_4_^3–^ in the HA with CO_3_^2–^ ions, which can be produced from gaseous carbon dioxide or be converted into carbonic acid under the alkaline condition [57]. In addition, owing to a high viscosity of the nanocellulose solutions, the Ca^2+^ ions were difficult to diffuse and thus first grasped by the OH^–^ ions, resulting in a partially remaining Ca(OH)_2_. In the second stage, the viscosity was decreased by an increase of temperature through the reaction, which induced the synthesis of HA to be surrounded with a large number of PO_4_^3−^ ions. 

**Figure 4:**
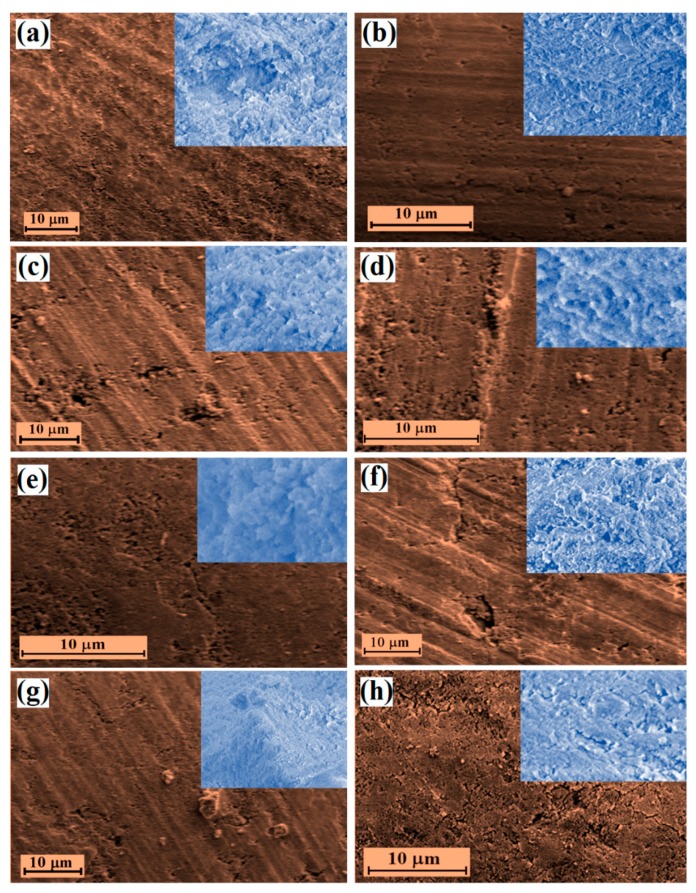
Representative SEM images of the surfaces and the cross-sections (presented in the inset) of the corresponding nanocomposites: (**a**) HATCNF10 prepared with 10 wt % of TCNF; (**b**) HACNC10 prepared with 10 wt % of CNC.; (**c**) HATCNF20 prepared with 20 wt % of TCNF; (**d**) HACNC20 prepared with 20 wt % of CNC; (**e**) HATCNF30 prepared with 30 wt % of TCNF; (**f**) HACNC30 prepared with 30 wt % of CNC; (**g**) HATCNF40 prepared with 40 wt % of TCNF; and (**h**) HACNC40 prepared with 40 wt % of CNC. The magnifications in all the figures are the same.

SEM images of the surface and the cross-section (in the inset) of all the synthesized and compressed HA-TCNF/CNC nanocomposites are presented in Figure 4. A rearrangement/reorientation of the components on the surfaces of the composites, being induced by the compression, as well as the CNF/CNC contents, was observed. A smoother and more uniformly distributed surface and cross-section can be observed in the case of the CNF-based composites compared to the CNC-based ones, where the lack of effective connections between smaller the HA–CNC particles was evident. It is obvious that the presence of the TCNF/CNC induced the nucleation of the HA and further modulated the kinetics and the morphology of the HA crystal growth [9,53] well as regulating the HA crystal structure, depending on the type of the nanocellulose and the available surface functional groups. As the crystallization was associated with the nucleation and the growth pattern and was highly related to the degree of saturation in the liquid phase, the representative HA–TCNF/CNC powders synthesized were agglomerated and irregularly shaped, compared to the eggshell-derived HA (Supplementary Figure S2). The powders were also larger in the samples containing CNC (Supplementary Figure S2c, HACNC20) compared to the TCNF-containing (Supplementary Figure S2b, HATCNF20) samples, which was most likely related to a better dispersibility of TCNF in a water solution. The effect of chemical interactions on the physical growth of HA could be also recognized, which was identified by a decrease of particle size for the composites synthesized in the presence of smaller and rod-like CNCs compared to that for the composites with fibrillated and a few micrometer long TCNFs. Besides, CNCs possesses an abundant amount of –OH groups, as well as some anionic OSO_3_^–^ groups, arising from strong sulphuric acid hydrolysis [61,62], both of which may trap Ca^2+^ ions through weak and specific coordination interactions (ion–dipolar forces) and nonspecific hydrogen bonding, as well as electrostatic interactions, and further attract PO_4_^3−^ ions from a precursor solution [9,63]. In the case of TCNF, electrostatic and coordination interactions may occur between Ca^2+^ ions and both OH^–^ and COO^–^ groups, leading to a stronger composite formation with more homogenously distributed components (as presented in Figure 4a,b). 

**Figure 5:**
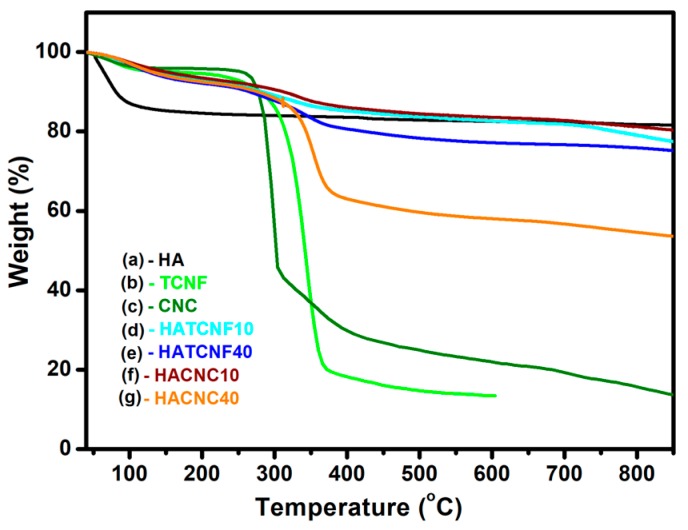
TGA thermograms of the pure HA (**a**), the TCNF (**b**), and (**c**) the CNC. TGA thermograms of the corresponding nanocomposites: (**d**) HATCNF10 prepared with 10 wt % of TCNF; (**e**) HATCNF40 prepared with 40 wt % of TCNF; (**f**) HACNC10 prepared with 10 wt % of CNC; and (**g**) HACNC40 prepared with 40 wt % of CNC.

The thermal stability of the samples was analyzed using TGA (Figure 5). The change of samples weight loss during the stepwise TGA was observed in the range between 40 and 850 °C, giving an indirection sight about the samples’ structures. As shown from the thermogram of the HA sample (Figure 5a), the weight loss was around 15.1% in the first temperature range of 30–167 °C, which was attributed to the loss of water and moisture trapped in the sample, and increased gradually by around 4% at temperatures up to 850 °C due to the decomposition of organic impurities and structural reformation. The thermogram of the TCNF (Figure 5b) shows an initial weight loss of around 4.5% between 40 and 300 °C due to an evaporation of adsorbed and loosely bound water, followed by its decomposition and the condensation of functional groups (–OH and –COOH) in the second phase (300–373 °C) [53,64,65] and the decomposition of cellulose chains in the third stage at temperatures up to 600 °C, with an overall weight loss of around 87%. All the thermograms of the nanocomposites show an initial first-phase weight loss at temperatures up to 300 °C similar to that of the pure TCNF (related to the evaporation of differently absorbed/bounded water), while a gradual reduction of weight loss can be observed in the region between 373 and 850 °C, resulting in a 19–21% weight loss in the case of the composites containing a lower percentage of nanocellulose (HACNC10 and HATCNF10, Figure 5e,c). The weight loss reduction was increased further to around 24% in the case of HATCNF40 (Figure 4d) and to around 46% in the case of the HACNC40 sample (Figure 5f), indicating the important influence of the composite structure, in which HA was formed around the nanocellulose to protect it. The latter was presumably also affected by the coverage homogeneity, which was found to be less in the case of the composite with highly concentrated CNC (HACNC40) compared to those of the other composites, including HATCNF40, thereby indicating the thermal instability of these samples. A higher weight loss reduction of the CNC-based composites may be also related to a lower intrinsic thermal stability of CNC due to the presence of remnant sulphate groups in the CNC [61] as well as a large number of free end chains of smaller CNC particles, which both facilitated the decomposition of the cellulose at lower temperature [62,66], consequently causing increases in the char yields of these hydrolyzed samples. 

### 2.2. Mechanical Properties of the Nanocomposites 

An alternative material that would be used for bone tissue regeneration should be mimicking the properties of the natural bone, and above all, it should possess enough stiffness and flexibility to be capable to resist deformations behavior at different loadings and displacements [2]. The mechanical properties of such a material, particularly fracture toughness, which defines how an implant will resist the cracking and is thus one of the most crucial safety issues of such a material, need to be balanced to be used for specific bone graft application. The fundamental relationship underlying these properties can be defined by many approaches (e.g., compression strength, compressive elastic modulus, hardness, and fracture toughness), which were also studied in our previous work [44] by using the compression technique and the Vickers indentation method. 

**Figure 6:**
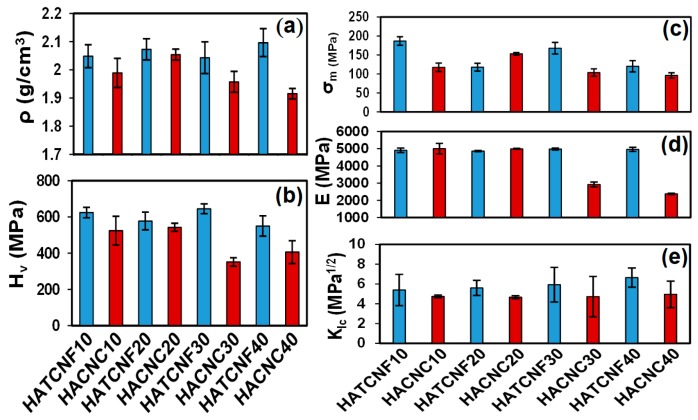
Mechanical properties of the corresponding nanocomposites: (**a**) density, (**b**) Vickers hardness, (**c**) compression strength, (**d**) elastic modulus, and (**e**) fracture toughness. HATCNF10 and HACNC10 are the samples prepared with 10 wt % of TCNF and CNC, respectively. HATCNF20 and HACNC20 are the samples prepared with 20 wt % of TCNF and CNC, respectively. HATCNF30 and HACNC30 are the samples prepared with 30 wt % of TCNF and CNC, respectively. HATCNF40 and HACNC40 are the samples prepared with 40 wt % of TCNF and CNC, respectively.

The results of the mechanical properties analyzed for the fabricated nanocomposites are presented in Figure 6. The density (Figure 6a) and the Vickers hardness (Figure 6b) of the composites prepared with different TCNF/CNC contents (10, 20, 30, and 40 wt %) were similar for all the TCNF-containing samples, while they decreased gradually by up to 45% and 50% in the case of the composites containing 30 and 40 wt % of CNC (samples HACNC30 and HACNC40, respectively), indicating a weaker CNC–HA interaction at a higher CNC content. This may be related to an irregular distribution of the nanomaterials (as discussed in relation to the SEM images presented in Figure 4), their poor interconnectivity, and/or the HA crystalline structure (as determined from the spectroscopic analysis, Figure 2 and Figure 3). Besides, the generally higher density (2.09–2.04 g·cm^–3^) and the higher hardness (550–640 MPa) of the HATCNF-based composites compared to those of the HACNC ones (density: 2.04–1.9 g·cm^–3^ and hardness: 542–405 MPa), with a small decline only for the HATCNF40 composite, are also in good agreement with those of the samples with a bulk structure analyzed in the previous section. The compression strength (Figure 6c) and the elastic modulus (Figure 6d) followed a similar trend, that is, both values were evidently higher and almost independent on the CNF content for the HATCNF composites (186–120 MPa and 5004 ± 308 MPa for the compression strength and the elastic modulus, respectively) compared to the HACNC ones, where they decreased to around 153–96 MPa and 2379 ± 39MPa, respectively, with more than 30 wt % of CNC addition. Consequently, the fracture toughness values, indicating a partial or complete break of the material, were higher for all the HACNF-based composites and increased with the increase of CNF content from 5.4 to 6.6 MPa^1/2^; compared to those of the HACNC ones with a range of 4.7–4.9 MPa^1/2^. The main reason, presumably, lies in the rigidity and the high crystallinity of the CNCs compared to the semicrystalline and flexible CNFs, as well as their interconnectivity/anchoring with the differently structured and distributed HA particles.

From the point of complex mechanical properties and the requirement to mimic the function of outer and dense cortical bone (with a mechanical strength between 100 and 230 MPa, an elastic modulus between 15 and 25 GPa, and a fracture toughness value between 2 and 12 MPa^1/2^) [67], the HATCNF composite prepared using 30 wt % of TCNF (with a compression strength of 167 ± 15 MPa, a Young’s elastic modulus of 4978 ± 55 MPa, and a fracture toughness of 5.9 ± 1.8 MPa^1/2^), seems to be the most suitable composite for bone regeneration applications.

### 2.3. Cytotoxicity of the Nanocomposites

**Figure 7:**
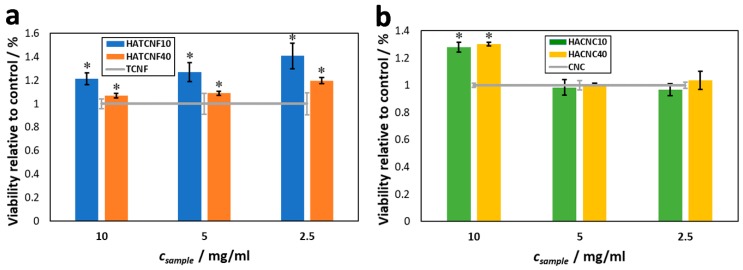
Viability of human osteoblast cells (hFOB) after 24 h of cultivation for 24 h at 37 °C and 5 wt % of CO_2_ for the composites: TCNF-based nanocomposite powders (HATCNF10 and HATCNF40 prepared with 10 and 40 wt % of TCNF, respectively) (**a**) and CNC-based nanocomposite powders (HACNC10 and HACNC40 prepared with 10 and 40 wt % of CNC, respectively) (**b**), relatively to the controls (the pure TCNF and CNC). * *p* ≤ 0.05.

The cytotoxicity testing using human bone-derived osteoblasts (hFOB) was performed on the HA–TCNF/CNC nanocomposite powders of different compositions (10 and 40 wt %) by evaluation of the potential cytotoxic/cytostatic response of the cells. According to the obtained viability measurement results presented in Figure 7, the nanocomposites induced no observable negative effect on the cell growth, as well as no zone of inhibition after 24 h of incubation in cell culture media, compared with the pure nanocellulose (the TCNF and the CNC). It can be seen immediately that the modification using HA with either nanocellulose type resulted in improved cell viabilities, regardless of the percentage of the HA in the composites. 

In the case of the TCNF-containing composites, the same results were also obtained for both of the prepared dilutions. The viabilities even increased a little bit with more diluted samples (Figure 7a), indicating the possible contact-inhibitory effect of the composites, which in larger quantities (lower dilutions) did not promote the cell growth as much as found for the diluted samples. With a decreased percentage of HA in the samples (e.g., in the case of HATCNF40; Figure 7a), the viabilities were also lower, indicating that the pure TCNF was less favorable for the growth of osteoblasts in its pure form. This observation is also in agreement with the measured viabilities for the control samples (the pure TCNF). 

Similarly, neither of the CNC-containing composites (Figure 7b) exhibited any toxic effect on the growth of osteoblasts, at least not on the level of the cell metabolism. However, surprisingly, the highest observed cell viability for the CNC samples was found for the base-undiluted samples, followed by those for the diluted samples. The latter, in both the cases, was almost indistinguishable from the control sample (the nonmodified CNC). Furthermore, contrary to the results found in the CNF samples, changing the percentage of the HA in the samples did not influence the overall cell viability at all. Considering both the mentioned results in regard to the nanocomposite type, we can presume that, in the case of CNC, the samples influenced the cell growth positively, when direct contact of cells with the suspended material occurred. Hence, after dilution, with fewer nanocomposites particles in the cell-culturing media, these particles did not come into contact with the cells with high rates, diminishing this positive effect on their growth. It can be concluded that the presence of the CNC (itself or as a composite) had a positive effect on the osteoblast growth, making it an interesting material, regardless of modification, for further studies towards its potential usage in bone tissue engineering. The observed positive influence of the CNC on cell growth was also in agreement with our previous studies [66].

## 3. Conclusions

The nanocomposites were prepared by synthesizing HA with either TCNF or CNC in situ and using eggshell-derived calcium and ammonium dihydrogen orthophosphates as phosphate precursors and a means of ultrasonication, and they were compressed further by a uniaxial high-pressure technique.

The contents of the TCNF and the CNC were shown to influence the composite structural and morphological properties strongly. Above all, HA formed crystallites with a crystal size that rendered the composite interconnectivity and further influenced the composites mechanical properties. In this sense, the composites prepared with relatively higher contents of semicrystalline and flexible TCNFs were found to demonstrate better compression strengths, elastic moduli, and fracture toughness, in the range of outer and dense cortical bone, than the CNC-based ones. Furthermore, regardless of the composite composition (either the used nanocellulose forms or the weight percentage), these composites did not induce cytotoxicity to human osteoblast cells. On the contrary, all the HA-containing composites improved the viability of the osteoblasts when compared to the pure CNCs or TCNFs. Based on all the above mentioned results, the prepared nanocomposites may be promising candidates for bone tissue regeneration in load-bearing applications, such as defects in large bone augmentation or grafts. These materials could be used either on their own or as part of other formulations, potentially improving their mechanical properties and/or osteointegration potential. 

## 4. Experimental 

### 4.1. Materials and Methods 

Eggshell was used as a calcium precursor; ammonium hydroxide (NH_4_OH), ammonium dihydrogen orthophosphate (NH_4_H_2_PO_4_), and hydrogen peroxide (H_2_O_2_) were received from Fisher Scientific, India. The CNFs (flexible and branched fibrils with 20–50 nm in width and several hundred micrometers in length) and the CNCs (rod-shaped particles with 5–20 nm in width and 150–200 nm in length) made from wood pulp were purchased from the University of Maine, the Process Development Center, USA. The TEMPO reagent, sodium bromide, sodium hypochlorite solution, ethanol (EtOH), and other chemicals were procured from Sigma-Aldrich and used as received.

### 4.2. Preparation of the HA–TCNF and HA-CNC Nanocomposites 

The preparation of CaO from the eggshell and its further conversion into Ca(OH)_2_, used as a calcium precursor for the preparation of the HA, was presented in our previous work [68]; the detailed preparation protocol was given in Supplementary Materials. TEMPO-mediated oxidation of the CNF was performed as previously reported elsewhere [40,69]; the detailed procedure was given in Supplementary Materials. In order to prepare the HA–TCNF/CNC nanocomposites with a varying TCNF/CNC composition, the prepared solutions of the precursors were mixed with 10, 20, 30, and 40 wt % of the TCNF and CNC suspensions, respectively, followed by their ultrasonication for 10 min to form homogeneous dispersions. The dispersed suspensions were stirred for another 10 min on a magnetic stirrer, followed by ultrasonication for 60 min, with an interval of 10 min to avoid overheating. The products (the HA and the HA–CNF/CNC composites) were then washed several times with deionized water, vacuum filtered, and kept for drying overnight in an oven at 60 °C. The dried products were ground in an agate mortar and pestle set to obtain fine powders, which were compressed at a pressure of 15 kN or 764 MPa by using a uniaxial compression technique (Smitweld 1405) to form cylindrical pellets and further characterized and studied. 

The samples were labeled related to the type and the weight percentage of each component: i.e., HATCNF10 (10 wt % of TCNF and 90 wt % of HA), HACNC10 (10 wt % of CNC and 90 wt % of HA), HATCNF20 (20 wt % of TCNF and 80 wt % of HA), HACNC20 (20 wt % CNC with 80 wt % HA), HATCNF30 (30 wt % of TCNF and 70 wt % of HA), HACNC30 (30 wt % CNC and 70 wt % HA), HATCNF40 (40 wt % of TCNF and 60 wt % of HA), and HACNC40 (40 wt % CNC and 60 wt % HA). 

### 4.3. Characterization of the Nanocomposites

The FTIR spectra over a region of 400–4000 cm^–1^ and a spectral resolution of 4 cm^–1^ were recorded at ambient conditions by a Perkin-Elmer spectrum one FTIR spectrometer (USA) with a Golden Gate ATR attachment and a diamond crystal. The spectra were carried out from accumulating 25 scans and with air spectrum subtraction performed in parallel as a background. The Spectrum 5.0.2 software program was applied for the data analysis. All the measurements are carried out in uplicate. 

The XRD analysis of the samples was performed using the D4 Endeavor diffract meter (Bruker AXS, Karlsruhe, Germany) with a Sol-X dispersive detector. The diffraction patterns were recorded using CuKα radiation at a voltage of 40 kV and a current of 30 mA. The range of 2θ was chosen from 5° to 80°, with a step size of 0.02 and a collection time of 3 s. The average crystallite size of the synthesized HA was estimated at 2θ of 25°, 32°, 39°, and 46° using the Scherrer equation [68]:

t_(*hkl*)_ = 0.9λ/B·cosθ_(*hkl*)_, (1)

where *λ* is the wavelength of the monochromatic X-ray beam (*λ* = 1.54056 Å for CuKα radiation), *B* is the full width at half maximum (FWHM) of the peak at the maximum intensity, *θ*_(*hkl*__)_ is the peak diffraction angle and satisfies Bragg’s law for the (*h k l*) plane, and *t*_(*hkl*__)_ is the crystallite size. 

The structure and the morphology of the samples were characterized by using a low-vacuum scanning electron microscope (SEM) (FEI Quanta 200 3D, USDA, Washington, D.C., United States), equipped with an energy dispersive X-ray spectrometer (EDXS) (Oxford INCA 350, Oxford, UK). The samples were sputter-coated with gold before being examined using the backscatter (BSC) mode and the secondary electron (SE) mode at different magnifications. High-vacuum field-emission SEM (FEI Sirion NC 400, USA, Lausanne, Switzerland) was used within a magnification range of 250–200,000 times.

TGA of the samples was performed under inert nitrogen conditions within a temperature range between 25 and 1000 °C and at a testing rate of 10 °C·min^−1^, using a PerkinElmer Pyres 1 thermogravimetric analyzer.

The mechanical properties (density, hardness, fracture toughness, compression mechanical strength, and compression elastic modulus) of the nancomposites were evaluated using 100 or 200 mg-weight cylindrically shaped pellets with a diameter of 5 mm, as reported in our previous study [44]. The pellets were prepared from the nanocomposite powders by compression methods under controlled conditions (force of 15,000 N and crosshead velocity of 0.05 mm·s^–1^) and displacements, measured by a tensile testing unit (TTU 2002) of the SMITWELD 1405 testing machine. The pellets with a weight of 100 mg were used to perform the Vickers hardness and fracture toughness tests, while the pellets with a weight of 200 mg were used to perform the compression tests. The hardness measurement was performed by a 6 kg load applied for 20 s using a standard Vickers diamond indenter with a 136° angle (Shimatzu HV2000 machine, Kyoto, Japan); indentation was followed by a scanning electronic microscope (FEI Sirion NC 400, Washington, D.C., United States, USA), where two diagonals of the indentation were measured. The compression strength was obtained as the maximum value of the applied stress, performed on the tensile testing unit 2002 of the SMITWELD 1405 testing machine. Young’s modulus was determined by the compression method. The linear elastic fracture toughness (*K_Ic_*) values of the composites were calculated with the stress intensity factor *ΔK*, at which a thin crack in the material began to grow, and measured in the unit of MPa·m^1/2^ or ksi·in^1/2^. Fracture toughness was achieved on a Vickers hardness machine ZWICK by indenting the Vickers’s diamond pyramid with an angle of 136° into the pellets. The model of fracture toughness was given in Supplementary Materials (Figure S1). Depending on the nature of the composites and the appearance of the crack (Palmquist/elliptic- or half penny-shaped), the fracture toughness was calculated by the half-penny and Palmquist models, respectively, according to the following equations [44,70]:

(2) $$K_{IC}=0.0089{\left(\frac{E}{H_{v}}\right)}^{\frac{2}{5}}\frac{P}{ac^{1/2}},$$

(3) $$K_{IC}=0.014{\left(\frac{E}{H_{v}}\right)}^{1/2}\frac{P}{c^{3/2}},$$

where *E* is the modulus (MPa), *H_v_* is the measured hardness (MPa), *P* is the applied load, and *c* is the radical crack length (mm). The densities of the composites were determined by weighing the mass of each pellet and measuring their geometric parameters by a Vernier caliper. The results presented are the mean values ± standard deviations of at least three independent measurements. Analysis of statistical significance (*p* ≤ 0.05) were performed by means of a one-way analysis of variance (ANOVA) using the SPSS software.

### 4.4. Cytotoxicity Testing

The cytotoxicity testing of the differently synthesized nanocomposites was performed using human osteoblast cells (hFOB 1.19, ATCC CRL 11372, ATCC, London, UK). Approximately 30 mg of the samples was suspended in 3 mL of the cell culturing media (Advanced Dulbecco’s modified Eagle’s medium, ADMEM, Gibco, MA, USA), supplemented with 5 wt % of fetal bovine serum (FBS, Gibco, USA). Before applying them to the cells, the samples were centrifuged at 1125 × *g* for 5 min to remove large agglomerates and sterilized under UV light for 30 min. The hFOB cells were applied to a P96 microtiter plate with a density of 10,000 cells per well, followed by incubation for 24 h at 34 °C and with 5 wt % of CO_2_. Subsequently, the as-prepared samples, as well as their dilutions in the ratios of 1:2 and 1:4 in the cell culturing media, were applied to the cells in four replicates. As the control, suspensions of the native CNF and CNC were used. These cell–sample combinations were incubated for 24 h at 37 °C with 5 wt % of CO_2_, before the cells viability was evaluated via the reduction reaction of the tetrazolium salt MTT (3(4,5 dimethylthiazolyl-2)-2,5-diphenyltetrazolium bromide) according to Mosmann [71] 

The obtained results are presented as means ± confidence intervals, calculated as ±$\;ts/\sqrt{x}$, where *t* is a Student’s *t*-distribution, *s* is the standard deviation, and *x* is the number of measurements. The analysis of statistical significance (*p* ≤ 0.05) was implemented using a one-way ANOVA test and Microsoft Office Excel 2016 (Microsoft, USA).

## Figures and Tables

**Table 1:** Elemental analysis, Ca/P ratio, and crystallite size of HA in different nanocomposites.

	HATCNF10	HATCNF40	HACNC10	HACNC40
C (wt %)	7.59 ± 0.71	10.10 ± 0.67	7.56 ± 5.82	14.39 ± 4.15
O (wt %)	49.66 ± 0.79	49.68 ± 2.46	46.56 ± 1.21	47.95 ± 0.98
P (wt %)	12.75 ± 0.26	13. 69 ± 0.72	14.16 ± 2.11	10.81 ± 1.65
Ca (wt %)	30 ± 1.10	26.52 ± 2.25	31.72 ± 4.71	26.84 ± 3.25
Ca/P ratio	2.35	1.93	2.24	2.48
HA crystallite size D_xrd_ (nm)	14.56	12.22	16.25	12.53

## Supplementary Materials

The following are available online at https://www.mdpi.com/2079-4991/10/1/25/s1, Figure S1: Model for fracture toughness measurement by the Vickers indentation method; Figure S2: Representative SEM images of (a) HA, (b) HATCNF20, and (c) HACNC20 powders; Figure S3: EDX spectra of respective nanocomposites.
