# Supplementary material for: Mechanical Properties and Cytotoxicity of Differently Structured Nanocellulose-hydroxyapatite Based Composites for Bone Regeneration Application
Source: Nanomaterials (Basel). 2019 Dec 20;10(1):25. doi: 10.3390/nano10010025 (PMC7022391; doi:10.3390/nano10010025)
Supplement: Supplementary file 1 [file nanomaterials-10-00025-s001.pdf]

Mechanical Properties and Cytotoxicity of Differently Structured Nanocellulose-hydroxyapatite Based Composites for Bone Regeneration Application

Vijay H. Ingole [1,2], Tomaž Vuherer [2], Uroš Maver [3], Aruna Vinchurkar [4], Anil V. Ghule [1,5] and Vanja Kokol [2,*]

[1] Department of Nanotechnology, Dr. Babasaheb Ambedkar Marathwada University, Aurangabad 431004, Maharashtra, India; mvt.vijay@gmail.com (V.H.I.); anighule@gmail.com (A.V.G.)
[2] Institute of Engineering Materials and Design, Faculty of Mechanical Engineering, University of Maribor, Smetanova ulica 17, Maribor SI-2000, Slovenia; vanja.kokol@um.si
[3] Institute of Biomedical Sciences and Department of Pharmacology, Faculty of Medicine, University of Maribor, Taborska ulica 8, Maribor SI-2000, Slovenia; uros.maver@um.si
[4] Department of Biophysics, Government Institute of Science, Dr. Babasaheb Ambedkar Marathwada University, Aurangabad 431004, Maharashtra, India; aruna.vinchurkar@gmail.com
[5] Department of Chemistry, Shivaji University, Kolhapur 416004, Maharashtra, India
* Correspondence: vanja.kokol@um.si; Tel.: +386-(0)2-220-7896

Preparation of hydroxyapatite nanoparticles

1.23 g of $Ca(OH)_2$ obtained from eggshells was mixed with 1.15 g of $NH_4H_2PO_4$, corresponding to the stoichiometric ratio of Ca/P = 1.67 as HA precursors. The mixture was ground in an agate mortar pestle, dissolved in 50 mL of deionised water, and then heated on a magnetic stirrer for 20 min to form the HA solution, followed by ultrasonication with 50 Hz for 1 h using an Ultrasonic bath machine-9L250N/D.T.C (PCI Analytics Pvt. Limited, India; 230 V AC) and heating further on a magnetic stirrer to evaporate the water.

Preparation of TEMPO-oxidised CNFs (TCNF)

The oxidation solution was prepared by dissolving 0.01475 g of TEMPO and 0.162 g NaBr in distilled water, and adding to 1 wt % of CNF dispersion. The NaClO solution (25 mL, 10%) was added to this slurry slowly and stirred at room temperature, and the pH of the solution was adjusted to 10 and kept constant for 3 h using 0.1 M HCl and 0.1 M NaOH. After stirring for the designated time, the oxidation was quenched by adding 7 mL of EtOH. The oxidized CNF was washed thoroughly with deionised water and weighed to measure the mass recovery ratios.

The sodium carboxylate groups in the TEMPO-oxidized cellulose nanofibers (CNFs) were further converted to free carboxyl group by ion-exchange treatment; 0.1% cellulose/water suspensions were adjusted to pH 2–3 with 0.1 M HCl at and left at rest for 1 h followed by washing thoroughly with water. The carboxylate content was evaluated by the potentiometric titration method, being increased from 0.3 ± 0.15 mmol g^{-1} for CNF to 1.63 ± 0.21 mmol g^{-1} for TCNF.

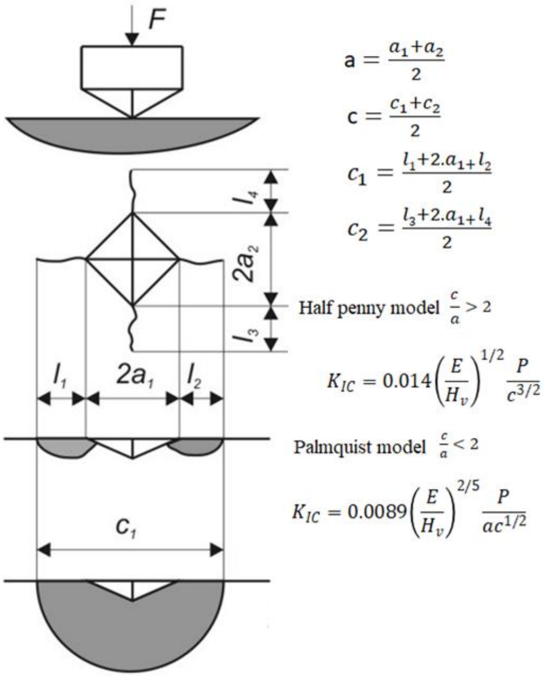

Figure S1. Model for fracture toughness measurement by the Vickers indentation method.

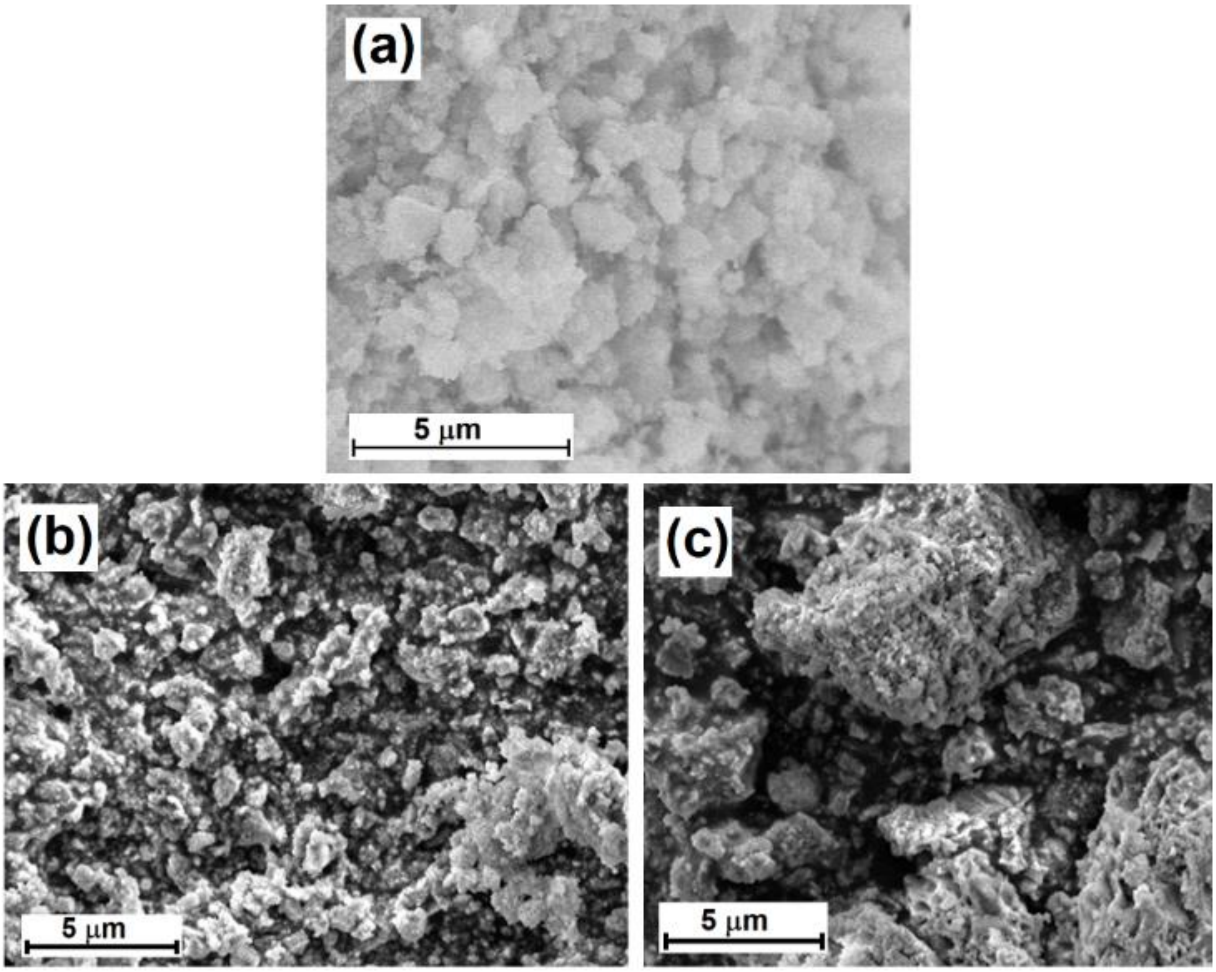

Figure S2. Representative SEM images of (a) HA, (b) HATCNF20, and (c) HACNC20 powders.

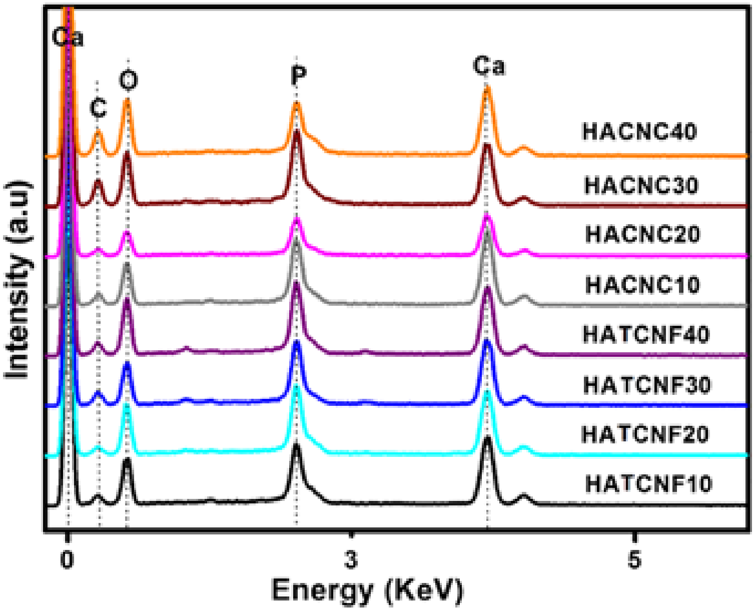

Figure S3. EDX spectra of respective nanocomposites.
